# The Old and the New—An Ellege

**DOI:** 10.1055/a-2411-7005

**Published:** 2024-09-27

**Authors:** Joon Pio Hong, Geoffrey G. Hallock

**Affiliations:** 1 Department of Plastic and Reconstructive Surgery, Asan Medical Center, University of Ulsan College of Medicine, Seoul, Republic of Korea; 2 Division of Plastic Surgery, St. Luke's Hospital, Sacred Heart Division, Allentown, Pennsylvania

**Figure:**
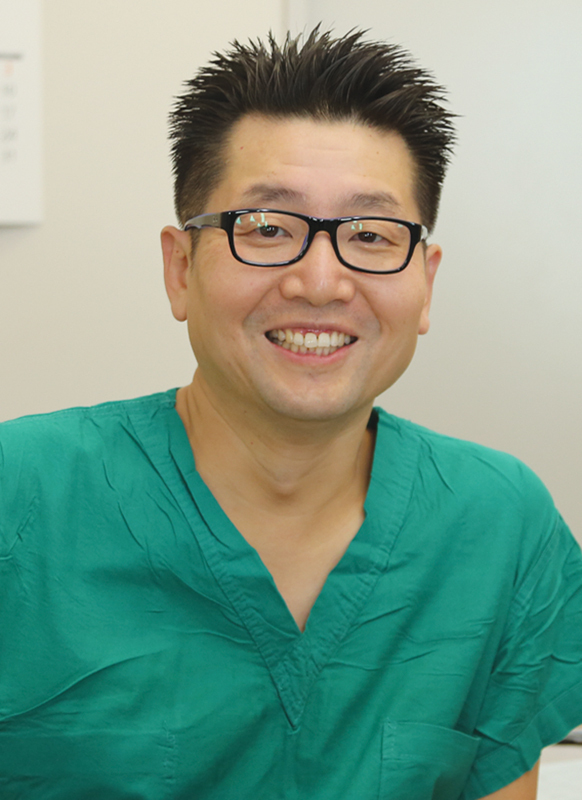
Joon Pio Hong: Editor-in-Chief

**Figure:**
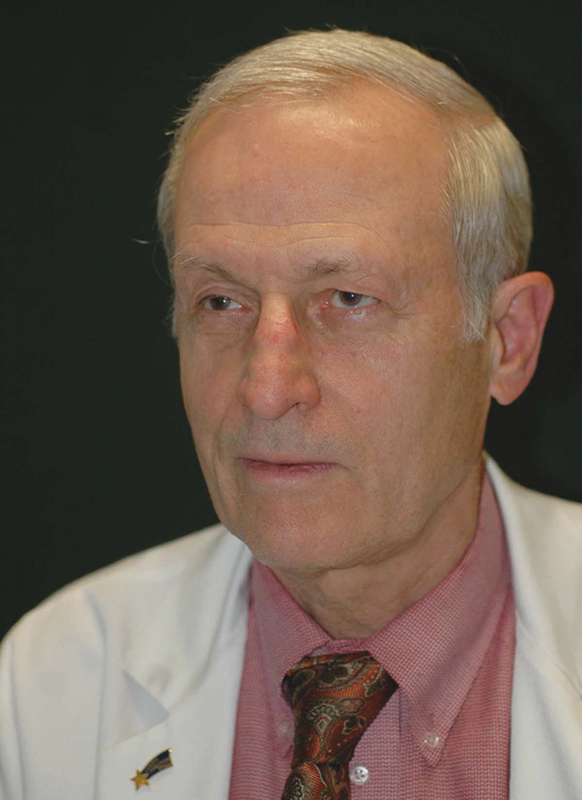
Geoffrey G. Hallock: : Associate Editor

*“The philosophies of one age have become the absurdities of the next, and the foolishness of yesterday has become the wisdom of tomorrow.”* –Sir William Osler 1

For eons, stories began in the same way—“Once upon a time…” and so does this story, as we reflect on how the past becomes the future; how “old” becomes “new” and “new” inevitably “old.” Once upon a time, there was no such dilemma. For generations, there existed the **oral culture** or **oral lore** where speech, song, or variation thereof was the only means to receive, transmit, or more importantly preserve what knowledge and traditions had been accumulated over time. 2 This phenomenon existed globally; and still does today, albeit in many places in parallel with the written word. Yet many dare to affix that condescending adjective “old” to this past behavior. After all, to be “old” is to be antiquated, archaic, obsolete, outdated, unwanted, or just plain weathered from mental or physical disabilities that so characteristically are the cause of the “uselessness of old age.” 3 To prove our assertion, did not Sir William Osler bid farewell to the Johns Hopkins University School of Medicine at the turn of the last century by stating “ *men older than 60 years should be retired* ,” and added that “ *men older than that age be chloroformed* .” 3 At the least, respect for old age was not deserved, and whomever or whatever was “old” should drown in oblivion.

So is “new” quite simply the converse of “old?” Must not “new” be the complete opposite of “old?” Chronologically, does “young” not replace their elder? Certainly, youthful energy exists unlimited to unleash its unimpeded consequences. To be “new” must be novel, modern, original, or innovative. 4 To rejuvenate implies to make “new,” whether to refresh, renovate, renew, restore, or even reincarnate. But heed Ralph Waldo Emerson, “ *old and new make the warp and woof of every moment. There is no thread that is not a twist of these two strands* .” 5 “Warp” and “woof” in weaving as not so risky a double entendre may refer to the crisscrossing of the threads, but can the textile of life be produced one without the other? Should we not conclude that “old” and “new” in whatever form are not disparate entities? Can one exist without being interwoven with the other? We concede that every rule has an exception, specifically the advent of microsurgical tissue transfers that so abruptly expanded the capabilities of the reconstructive surgeon. 6 Did not the preamble by Harry J. Buncke, Jr., correctly prophesy the future—“ *The successful transplantation of a block of composite tissue by reanastomosing the microvascular pedicle has untold experimental and clinical possibilities?* ” 7

But rarely do such dynamic events as the aforementioned occur. More often our evolution has not been a tsunami but rather an incremental progression, indeed minor alterations or modifications of the past. 8 There exists a “yin and yang,” where the opposing forces of “old” and “new” maintain a virtually symbiotic relationship that interconnects and balances each other. Witness the dual usage of these very same words within the titles of our literature, in the description of “new” flaps, 9 10 11 “new” techniques, 12 “new” means to learn and teach, 13 and ever-changing philosophical concepts. 14 15 16 Goldwyn said all this well, to survive and flourish we must find “new wine in old bottles.” 17

And change we must, as Sir William Osler once said, “ *everywhere the old order changes, and happy they who can change with it* .” 18 The myth of Plato long ago related to us that Socrates once criticized the invention of writing, as this would weaken the power of memory and critical thinking capabilities so important in the oral culture of his time. 19 Will in our so modern time omnipresent digital media and large language models raise the same fears? Will artificial intelligence become an alternative intelligence? Will robots so armed eliminate the need for “ *Homo sapiens* ” themselves? 20 As guardians of our specialty, we must persevere against all these odds to ensure progress, not forgetting as Confucius bided us to “ *acquire* ***new*** *knowledge whilst thinking over the* ***old*** , *and you may become a teacher of others* .” 21 Our knowledge so nominal is stored within our literature, and change depends on all of us. We must not fear to actively participate even if no more than to resurrect the past as that could become the future. Remember again the wisdom of Constantian, “ *old articles never die; some mercifully should, some shouldn't, and some should re-emerge in a new corpus* .” 14 But if never written, it was never done.

Every story has an ending, and ours returns to the Lehigh Valley where the young student, Lew Jae Duc, renewed himself to become of all things the father of Korean Plastic Surgery. 22 There can be found in the oral lore of the Indigenous Pennsylvania “ *Dutch* ” here our sentiments recapitulated, “ *too soon old, too late schmart!* ” Time flies. And “new” soon becomes “old.”
